# Evaluating the frequency of successful guidewire crossing through a complex lesion in coronary artery disease patients having chronic total occlusion

**DOI:** 10.12669/pjms.38.5.4770

**Published:** 2022

**Authors:** Waheed Akhtar, Syed Tehseen Shah, Shahzad Hasrat, Waqar Mustafa

**Affiliations:** 1 Waheed Akhtar, MBBS, FCPS., Abbas Institute of Medical Sciences, Muzaffarabad Azad Jammu and Kashmir, Pakistan; 2 Syed Tehseen Shah, MBBS, FCPS., Abbas Institute of Medical Sciences, Muzaffarabad Azad Jammu and Kashmir, Pakistan; 3 Shahzad Hasrat, MBBS, FCPS., Abbas Institute of Medical Sciences, Muzaffarabad Azad Jammu and Kashmir, Pakistan; 4 Waqar Mustafa, MBBS, FCPS., Abbas Institute of Medical Sciences, Muzaffarabad Azad Jammu and Kashmir, Pakistan

**Keywords:** Angioplasty, Chronic Total Occlusion, Coronary Artery Disease, Percutaneous Coronary Intervention, Stents and J-CTO score

## Abstract

**Objectives:**

To determine the frequency of successful guidewire crossing through chronic total occlusion (CTO) in patients having a J-CTO Score = 2 (difficult lesion).

**Methods:**

A prospective, cross-sectional study was conducted at the Armed Forces Institute of Cardiology (AFIC) in Rawalpindi. Patients with high calcium score on CT-angiogram were sent for elective coronary angiogram out of which patients diagnosed with chronic total occlusion (CTO) were selected and J-CTO Score was assessed. Those with a J-CTO score = 2 (difficult lesion) were enrolled for percutaneous coronary intervention (PCI). Guidewire that can cross the lesion within 30 minutes was considered successful.

**Results:**

A total of 158(95.8%) cases had successful guidewire crossing, while in 7(4.2%) patients, the procedure was unsuccessful. No significant association between the success rate of guidewire crossing and age (p = 0.21). Furthermore, there was no statistically significant relationship between guidewire crossing and LV function (p = 0.559) i.e. 32.2% and 42.9% of those with LV function between 25-35% had successful and unsuccessful guidewire crossing, respectively. While 67.7% and 57.1% patients having 36-65% LV function were observed having successful and failed PCI, respectively.

**Conclusions:**

The success rate of guidewire crossing through CTO in patients having a J-CTO Score =2 (difficult lesion) is acceptable so J-CTO score can be considered for difficulty grading of the lesion before intervention to prevent complications and success rate of PCI.

## INTRODUCTION

Cardiovascular disorders, particularly coronary artery diseases, contribute to almost one-third of the world-wide mortality rate.^1^ However, the exact figure is still unknown as a subset of the patients does not go through the diagnostic process.^1^ In most cases, chronic total occlusion (CTO) is a common factor in patients with coronary artery disease (CAD).^2^ CTO is prevalent among CAD patients who undergo angiography, and an estimated value suggests that it is around 18 to 30%.^3^

Percutaneous coronary intervention (PCI) is a minimally invasive procedure and potentially beneficial process in lowering the intensity of the symptoms and reduces mortality.^4^ PCI is considered a relatively influential determinant to refer the patients to either a coronary artery bypass grafting (CABG) or treatment with the only medications.^5^ Several effective interventions techniques treat CAD patients with CTO like parallel, retrograde, and antegrade wire techniques. Still because of the complications most of the patients were treated with medications.^6^-^8^

It will be of great value to evaluate the probability of successful guidewire crossing through CTO to identify the success rate of revascularization and complications. The J-CTO score is the reliable tool that can be applied in CTO patients for grading the lesion for PCI. The different morphological features of CTO lesions like entry shape, bending, calcification and length of the occlusion to objectify the procedural difficulty in performing PCI.^9^ This score was formulated in Japan and has been used as an effective predictive tool for determining the success rate of PCI.^9^-^11^

As the technology progressed, many techniques like computed tomography (CT), coronary angiogam are excellent diagnostic tools for accurately predicting the difficulty in performing an intervention in patients with CTO. But J-CTO scoring is an easy and reliable method of assessing the difficulty of lesions and time required for crossing guidewire.^12^-^14^ So by using J-CTO Score this study aims to categorize CTO lesions, predict duration and success rate of PCI and planning referral to experienced centers in interventional techniques, and provide a safe and effective intervention method for cardiologists to select patients with CTO suitable for successful PCI.

## METHODS

This prospective, cross-sectional study was conducted at the Armed Forces Institute of Cardiology (AFIC), Rawalpindi, using a non-probability consecutive sampling technique. Sample size was calculated using WHO sample size calculator keeping confidence level (1-α) = 0.95, anticipated population proportion (P) =0.938 and absolute precision required (d) = 5%. The study was conducted between September 30, 2017, and March 30, 2018 and ethical approval was obtained from the ethics committee of Armed Forced Institute of Cardiology and National Institute of Heart Disease (Reference no: AFIC-IERC-SOP-15; Dated 5^th^ October 2016). A written informed consent was obtained from study participants before inclusion.

All patients between 20 to 70 years of age, having high calcium score on CT-angiogram were sent for elective coronary angiogram, out of which those diagnosed with chronic total occlusion (CTO) were selected and J-CTO Score was assessed. Patients having J-CTO score = 2 (difficult lesion) were included for PCI.

Further patients having angina of any severity undergoing elective coronary angiogram at AFIC and diagnosed as having CTO lesions in one or more coronary arteries were also enrolled. Patients undergoing primary PCI for acute total or partial occlusion, patients with left main system disease, severe LV dysfunction EF < 20%, diffuse disease involving multi-vessels and patients having J-CTO Score > 2 were excluded from the study. Interventional cardiologists experienced in doing PCI of CTO lesions performed PCI and Guidewire that crossed the lesion within 30 minutes was considered successful.

Frequency and percentage were calculated for qualitative variables like gender and guidewire crossing. Mean, and standard deviation was calculated for quantitative variables like age of patient and LV function. Effect modifier like age, gender, and LV function was controlled by stratification. Post-stratification was done by using the chi-square test, and a p-value ≤ of 0.05 was considered significant. The data was analyzed using SPSS version 21.0.

## RESULTS

The mean age of patients was 49.52 ± 11.06 years, with a minimum age of 20 years and maximum age of 70 years. Further stratification according to age is shown in Table-I. The mean LV function was 41.88 ± 10.42 % with a minimum of 25% and a maximum of 60%. There were 54(32.7%) patients who had LV function as 20-35%, and 111(67.3%) patients had LV function as 36-60%.

**Table I:** Frequency distribution of Successful Guidewire with respect to age groups and LV function.

Variables		Successful Guidewire		Total

		Yes (n=158)	No (n=7)	
Age groups	20-40 years	29(18.4%)	0(0%)	29(17.6%)
	41-70 years	129(81.6%)	7(100.0%)	136(82.4%)
LV function	25-35%	51(32.3%)	3(42.9%)	54(32.7%)
	36-65%	107(67.7%)	4(57.1%)	111(67.3%)

A total of 158(95.8%) cases had successful guidewire crossing i.e. 29 patients (20-40 years) and 129 patients (41-70 years), while in 7(4.2%) patients, the procedure was unsuccessful and all of these were 41-70 years old. No significant association was found when the frequency of successful guidewire crossing was analyzed with age (p = 0.21).

When gender was analyzed in relation to guidewire crossing, no statistically significant association was observed (p = 0.076). Out of 100 males, 98 had successful guidewire crossing, while out of 65 females, 60 had a successful outcome. Similarly, there was no statistically significant relationship between guidewire crossing and LV function (p = 0.559) (Table-I).

## DISCUSSION

CTO patients have multiple factors that contribute to worsening of the condition like up-regulation of immunologic and inflammatory markers, endothelial dysfunction, accumulation of lipids, fats, abnormal growth of smooth muscle cells, presence of calcium and neuro-vascularization.^15^-^17^ The data from National Heart, Lung and Blood Institute (1997-1999) suggest that these lesions are more frequent in the right coronary artery as compared to the left artery.^18^

In the last two decades, there is an evident advancement in coronary interventions, especially in relation to CTO-PCI that were previously considered being a less accurate determinant of the integrity of the lesion, but later many technological advancements have been made by the time. One of them is the design of the wire and different techniques used that has high crossing power with the least collateral damage. There are four primary techniques used to perform CTO-PCI. The first one is antegrade wire escalation, antegrade dissection and reentry, retrograde wire escalation and last but certainly not least retrograde dissection and reentry. To perform these techniques and interpret the results with utmost accuracy, skilled and experienced professionals or operators are required.^19^ As a matter of fact, the intervention’s success rate not only relies on the skills and handling of the operator but also on some anatomical features of the lesion such as type of calcification, length, etc. However, skilled operators reduce the probability of missing the mark. According to the guideline for coronary artery interventions provided by the American College of Cardiology/American heart association, CTO-PCI resides in class II a. The instructions provided by the American Heart Association states that PCI is reliable in CTO patients with proper indications and seemly anatomy when a skilled operator performs the task and patient is symptomatic besides guideline directed medical therapy.^20^

In our study, a total of 158(95.8%) cases had successful guidewire crossing. A similar study reported an overall success rate of 91.5%, which is comparatively low than that observed in the present study. A study was conducted in different experience centers of the United States that includes 650 consecutive patients who underwent CTO-PCI during the time span from 2011 to 2014. The researcher of the study classified the lesions according to the multicenter CTO registry of Japan. Antegrade wiring techniques were used for simple lesions, but as the difficulty level of the lesion rises, retrograde wiring techniques seem to be more effective as a predictive measure.^18^

A Korean study reported that there was no difference in the adjusted risks of myocardial infarction and death among the patients who experienced successful vs. failed PCI attempts i.e. there are no survival benefits intact. But there was a significant decline in the risk of target vessel revascularization and need for CABG among those with a successful PCI attempt.^21^ Over time, multiple studies were performed, which reflects similar results to support the efficacy of CTO-PCI procedure and application of J-CTO score before PCI to predict success rate and to prevent complications.^22^,^23^ Nombela et al., investigated the performance of the J-CTO score for predicting procedure complexity and success in an independent contemporary cohort. A total of 209 consecutive patients who underwent CTO recanalization by a high-volume operator were included. Clinical and angiographic data were prospectively collected. Clinical and angiographic differences were noted between the original and studied cohort. The mean J-CTO score was 2.18 ± 1.26, and successful guide wire crossing within 30 minutes and final angiographic success were 44.5% and 90.4%, respectively.^22^

### Limitations of the Study:

There are several associated limitations of the present study that must be recognized. First, potential limitation was inability to assemble the information regarding cardiovascular risk factors (smoking, family history of CAD, hypertension, diabetes, dyslipidemia and obesity) and hence it is not included in the present study findings. Secondly the clinical outcomes weren’t assessed. Furthermore, the success of guidewire was studied only with respect to age groups and LV function, while gender, smoking status, family history of CAD, and presence of comorbidities might also alter the overall success rate associated with PCI.

## CONCLUSION

A high frequency of successful guidewire crossing through CTO in patients having a J-CTO Score = 2 (difficult lesion) in the present study suggests that J-CTO score is effective and can be utilized for difficulty grading of CTO lesions and procedural success rate to avoid complications. Further large-scale studies are recommended in extension, to study the safety and clinical outcomes of PCI.
